# Tuberculosis combined with Burkitt lymphoma in a kidney transplant recipient: A case report and literature review

**DOI:** 10.1097/MD.0000000000033671

**Published:** 2023-05-05

**Authors:** Jian-Nan Hu, Mu-Qing Yu, Li-Juan Hua, Chen Bao, Qian Liu, Chao Liu, Zi-Ling Li, Xi Wang, Shu-Yun Xu

**Affiliations:** a Department of Respiratory and Critical Care Medicine, Key Laboratory of Pulmonary Diseases of Health Ministry, Tongji Hospital, Tongji Medical College, Huazhong University of Science and Technology, Wuhan, PR China; b Institute of Pathology, Tongji Hospital, Tongji Medical College, Huazhong University of Science and Technology, Key Laboratory of Pulmonary Disease of Ministry of Health of China, Wuhan, PR China.

**Keywords:** Burkitt lymphoma, kidney transplantation, post-transplant lymphoproliferative disorder, pulmonary tuberculosis, tuberculosis

## Abstract

Tuberculosis (TB) and post-transplant lymphoproliferative disorder are serious complications affecting the long-term survival of kidney transplant recipients (KTRs). Both of complications have overlapping clinical symptoms, signs, and high similar imaging presentation, which make early clinical diagnosis challenging. In this paper, we reported a rare case of post-transplant pulmonary TB combined with Burkitt lymphoma (BL) in KTR.

**Patient concerns:**

A 20-year-old female KTR presented to our hospital with abdominal pain and multiple nodules throughout the body.

**Diagnoses:**

TB is diagnosed based on the lung histopathology showed fibrous connective tissue hyperplasia with number of chronic inflammatory changes, localized necrosis, granuloma formation and multinucleated giant cells were seen in the lung tissue. Moreover, lung histopathology specimen tested positive for TB gene. TB The culture for tuberculosis was positive. BL was diagnosed as metastatic after completion of liver and bone marrow biopsy.

**Interventions:**

After an early diagnosis of TB, the patient received intensification of anti-tubercular therapy. Because the patient was diagnosed with BL, rituximab, cardioprotection, hepatoprotection and alkalinization of urine were added.

**Outcomes:**

After an early diagnosis of TB, the patient received anti-tubercular therapy and her clinical symptoms and imaging manifestations improved. After the diagnosis of BL was made, the patient’s condition progressed rapidly, followed by multi-organ damage and died 3 months later.

**Lessons:**

Therefore, in organ transplant patients, who present with multiple nodules and normal tumor markers, they should be alerted to the possibility of concurrent TB and post-transplant lymphoproliferative disorder, and perfect tests such as Epstein–Barr virus, β2-microglobulin, lactate dehydrogenase, γ-interferon release test and Xpert Mycobacterium TB/rifampicin test and perform early lesion site biopsy to clarify the diagnosis with a view to improving the prognosis.

## 1. Introduction

The best option for treating end-stage renal failure is a kidney transplant, but those who receive one must take anti-rejection medications for the rest of their lives, and long-term glucocorticoids and immunosuppressive drugs compromise the immune function of the body, leading to easily complicated infections and tumors. With a global co-prevalence of 2.51% and considerably higher in underdeveloped nations, tuberculosis (TB) is the most prevalent infectious complication following kidney transplantation and is thought to be a significant issue influencing the long-term survival of kidney transplant recipients (KTRs).^[1]^ In addition to TB, post-transplant lymphoproliferative disorders (PTLDs) are the second most common malignancy in adult KTRs.^[2]^ Among PTLDs, adult Burkitt lymphoma (BL) is rarely reported in solid organ transplant (SOT) patients.^[3,4]^ To date, cases of complicated TB or PTLDs have been reported separately in KTRs, but the coexistence of both TB and BL in KTRs is rarely reported and have brought difficulty in diagnosis and treatments. In this article, we reported a rare case of combined TB and BL in a KTR and reviewed the literature.

## 2. Case report

The patient, a 20-year-old female, was admitted to the hospital on June 23, 2020, with “abdominal pain over 1 month.” She had received kidney transplantation for chronic renal failure on August 20, 2013, and October 1, 2019. Routine immunosuppressive therapy was performed after transplantation. She was diagnosed with pulmonary TB in 2018 and improved after regular anti-tubercular therapy (ATT).

On admission, the patient presented with abdominal pain from the upper abdomen to the umbilicus, increased abdominal girth, more night-sweat and shortness of breath. Physical examination showed the mild tenderness in the right hypochondrium and the lower hepatic border was located 1 transverse finger below the umbilical level. Superficial lymph nodes, shifting dullness, cardiac and respiratory sounds were negative.

Laboratory test showed elevated aspartate transaminase (41 U/L), alkaline phosphatase (352 U/L) and gamma-glutamyl transferase (932 U/L), and reduced pre-albumin (<80 mg/L) and lactate dehydrogenase (LDH) (104 U/L). Other serum biochemicals (blood count, renal function, hepatitis antibodies, and tumor markers) were normal. Besides, Tacrolimus (FK506) was 6.4 ng/mL.

**Figure 1.**
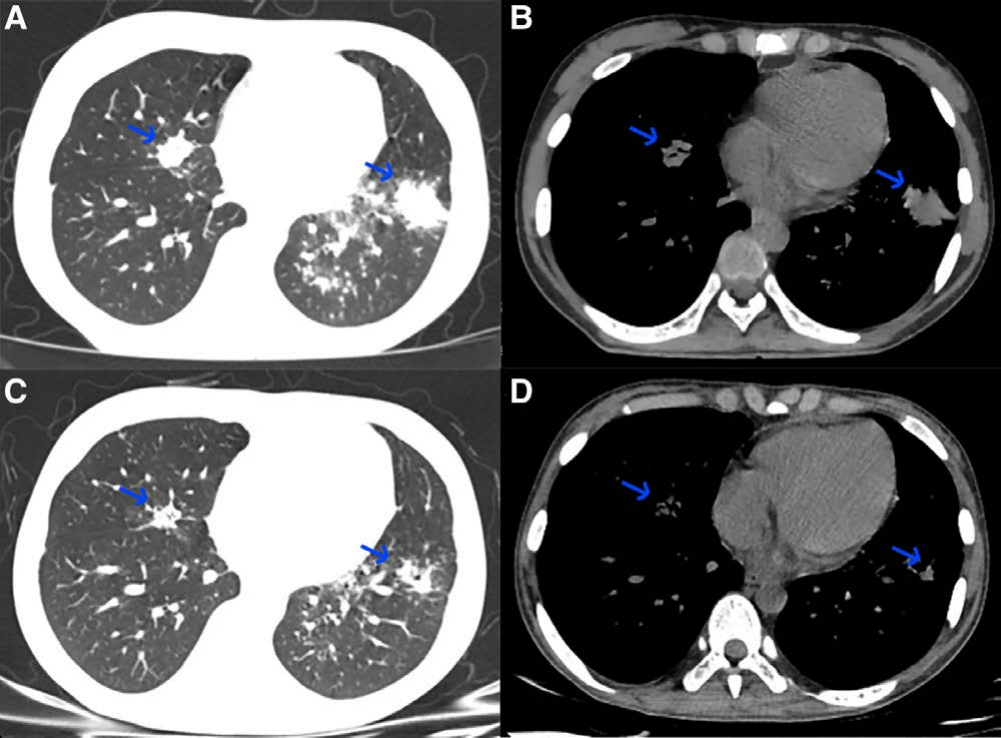
CT images of the chest before and after ATT. (A and B) CT of the lungs before ATT, with the 2 largest nodules in the lungs of 18 × 14 mm (right lung) and 30 × 22 mm (left lung). (C and D) CT of the lungs after ATT, with the 2 largest nodules in the lungs reduced to 14 × 9 mm (right lung) and 18 × 10 mm (left lung). ATT = anti-tubercular therapy, CT = computed tomography.

**Figure 2.**
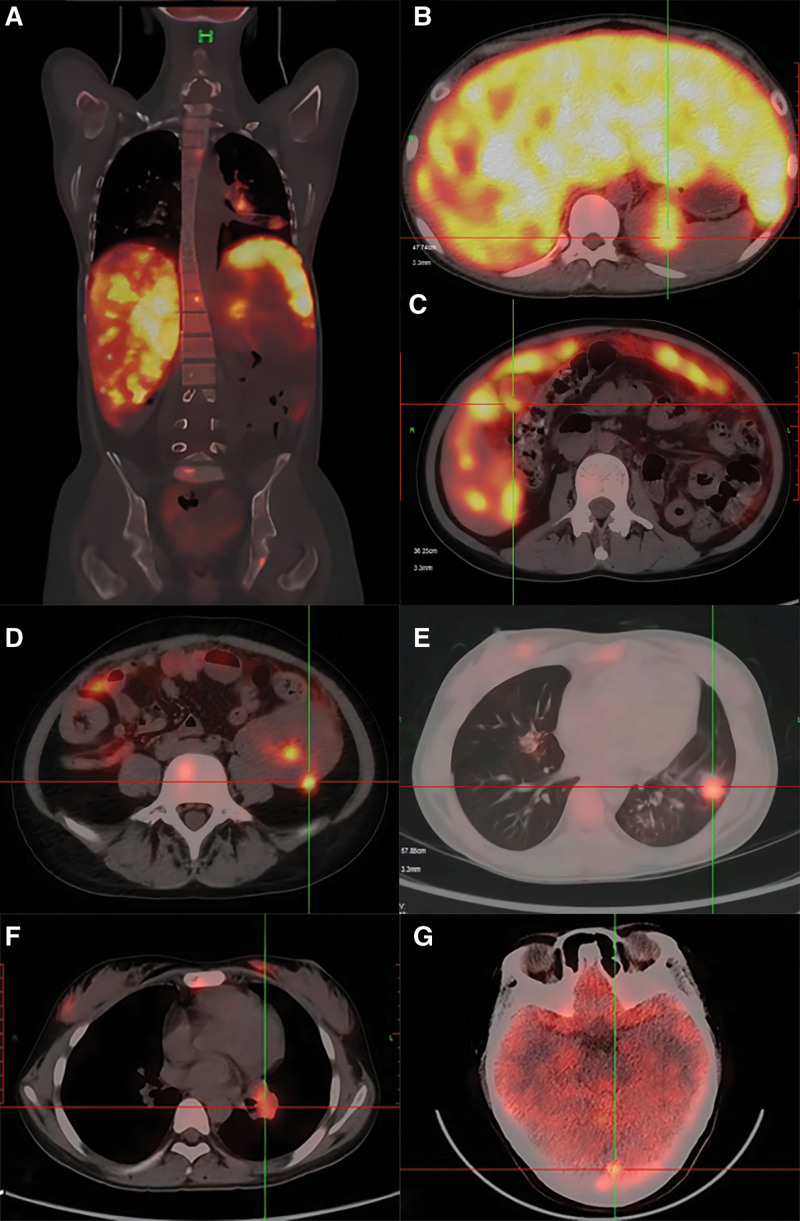
PET-CT images. (A) Showing multiple hypermetabolic lesions throughout the body. Increased and enlarged peritoneal and retroperitoneal lymph nodes with partially elevated metabolism; multiple elevated bone metabolism throughout the body (SUV_max_ 5.7). (B) Heterogeneously elevated metabolism in the liver (SUV_max_ 13.2), thickened gallbladder wall with elevated metabolism (SUV_max_ 8.3). (C) Locally elevated metabolism in the spleen (SUV_max_ 6.2) and splenic hilar nodule (25 × 15 mm). (D) Transplanted kidney localized metabolic increase (SUV_max_ 10). (E and F) left lower lung nodule (24 × 16 mm), metabolic increase (SUV_max_ 4.4), mediastinum and left hilar (SUV_max_ 6.1). (G) hypothalamic area, left occipital lobe metabolic increase (SUV_max_ 4.2), the above considered possible neoplastic lesions. SUV = standard uptake value, PET-CT = positron emission tomography-computed tomography.

**Figure 3.**
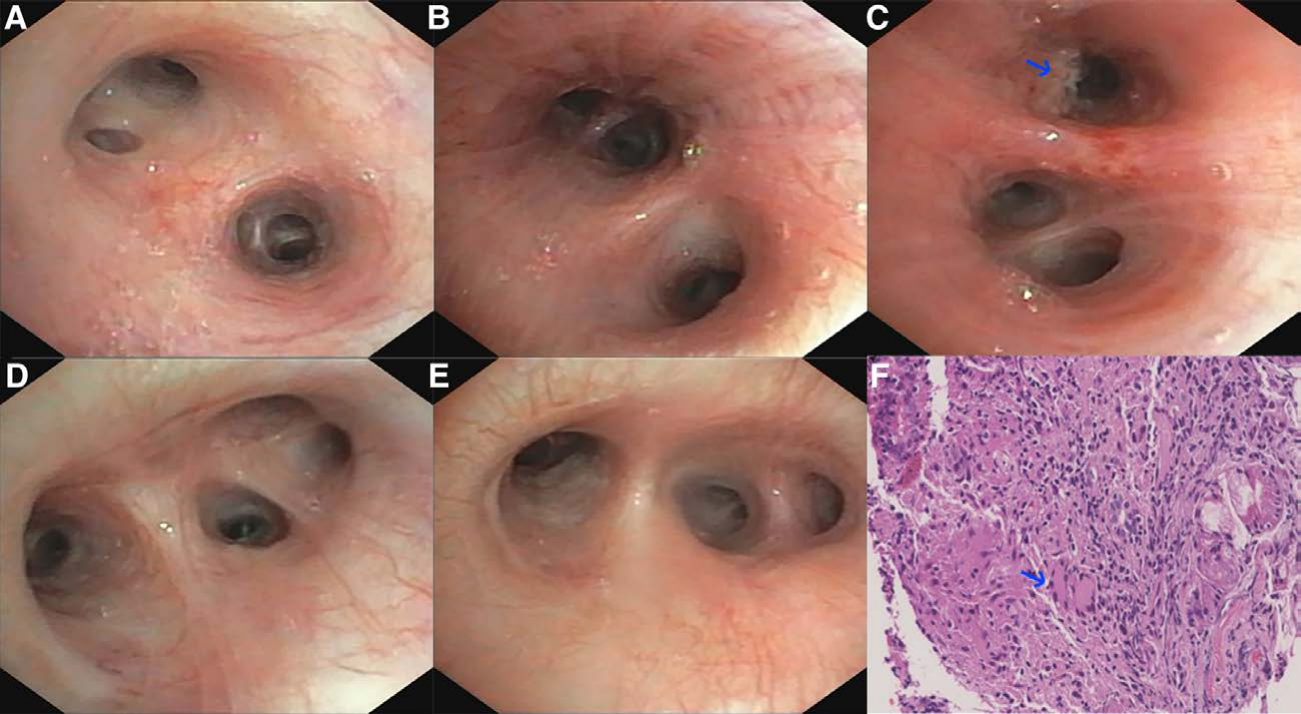
Bronchoscopy shows hypertrophic mucosal erosion in the anterior segment of the left lower lobe with histopathological findings suggestive of necrosis and granuloma formation. (A, B, D, and E) No significant abnormalities were seen in the left upper lobe, left lower lobe, right upper lobe, and right middle and lower lobe. (C) Limited elevation of the mucosa of the bronchial opening in the anterior basal segment of the left lower lobe with an uneven surface (arrow), swiped over here for examination. (F) HE staining (left lower lobe bronchial lesion) Microscopically, fibrous connective tissue hyperplasia with numerous chronic inflammatory changes, histiocytosis, localized necrosis, granuloma formation and multinucleated giant cells were seen in the lung tissue (F), and occasional multinucleated giant cells (shown by arrow in F). F: ×200.

Abdominal ultrasound showed multiple hypodense nodules in the liver and spleen with the largest hypoechoic area in the liver of 37 × 9 mm. Chest computed tomography (CT) showed multiple nodules and plaques in both lungs and enlarged mediastinal lymph nodes (Fig. 1A and B). Positron emission tomography-CT indicated multiple hypermetabolic lesions throughout the body (Fig. 2A–G). Endobronchial ultrasound-guided transbronchial needle biopsy of mediastinal lymph nodes was normal. Bronchoscopy shows that no significant abnormalities were seen in the left upper lobe, left lower lobe, right upper lobe, and right middle and lower lobe (Fig. 3A, B, D, and E), but a limited mucosal elevation with an uneven surface was seen in the anterior basal segment of the left lower lobe bronchial opening (Fig. 3C), where mucosal biopsy and lavage was performed. Lung histopathology showed fibrous connective tissue hyperplasia with number of chronic inflammatory changes, localized necrosis, granuloma formation and multinucleated giant cells were seen in the lung tissue (Fig. 3F). Moreover, lung histopathology specimen tested positive for TB gene. The culture for TB was positive. To further elucidate the nature of hepatic occupancy, liver puncture was proposed. However, high risk of hepatic puncture lead by the diffuse intrahepatic lesions prevented family from consent with this manipulation temporarily.

After diagnosis of pulmonary TB, triple combination ATT (isoniazid 0.3 g qd iv drip, rifampicin 0.45 g qd po, ethambutol 0.75 qd po) was used. However, the patient developed fever up to 39.3 ℃ with bacterial and fungal infections (−) and was considered to have symptoms of TB toxicity after ATT. After the intensification of ATT, with additional levofloxacin (0.6 g qd) and linezolid (0.6 g q12h), and an increase in hormone dose (the patient was maintained on long-term anti-rejection therapy with prednisone 5 mg/d, then increased to 15 mg/d), the body temperature gradually normalized. After 28 days of ATT, the patient’s symptoms such as abdominal pain and fever improved significantly. Physical examination also showed a decreasing abdominal circumference from 82 to 72 cm and liver volume. Liver and kidney function tests: ALT, aspartate transaminase, Ur, and Cr were normal; alkaline phosphatase was 127 U/L, gamma-glutamyl transferase 315 U/L and LDH was 100 U/L. The hypoechoic areas of the liver and pulmonary nodules were smaller than before (Fig. 1C and D).

**Figure 4.**
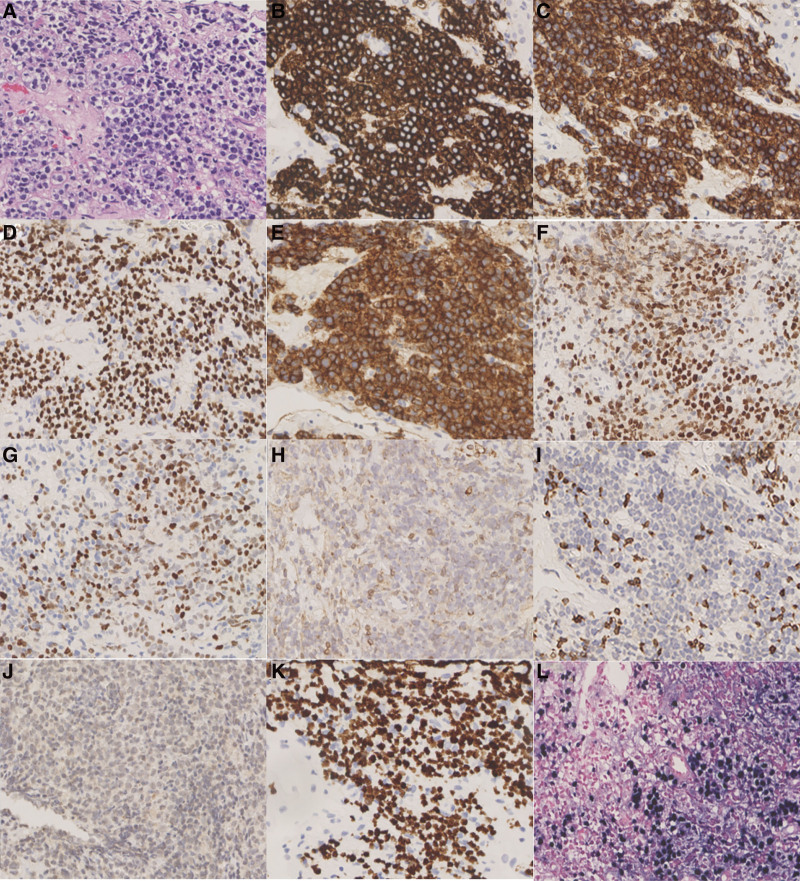
Liver tissue puncture pathology suggestive of Burkitt lymphoma. (A) Liver HE staining results: numerous proliferating lymphocytes are seen. Immunohistochemistry: (B) CD20 (+), (C) CD19 (+), (D) PAX5 (+), (E) CD10 (+), (F) BCL-6 (+), (G) MYC (~40% +), (H) BCL-2 (−), (I) CD3 (−), (J) TdT (−), (K) Ki67 (LI ~100%). In situ hybridization: (L) EBER CISH (+). A–L: ×400.

After the gradual stabilization of symptoms, a liver biopsy was performed to further clarify the nature of nodules. Pathology showed the PTLD (monotypic B-cell type). Liver HE staining results: numerous proliferating lymphocytes are seen (Fig. 4A). Immunohistochemistry results (Fig. 4B–L) was consistent with the BL: tumor cells CD20 (+), CD19 (+), PAX5 (+), CD10 (+), BCL-6 (+), MYC (~40% +), BCL-2 (−), CD3 (−), TdT (−), Ki67 (LI ~100%). In situ hybridization showed EBER CISH (+). Histochemical staining showed that acid-fast staining, periodic acid Schiff stain and methenamine silver stain were all negative. Also, the liver pathogenic detection was negative for acid-fast staining, bacterial, fungal and TB cultures and MTB genetic tests. Serum β2-microglobulin (β2-MG) was elevated at 4.74 mg/L with plasma Epstein–Barr virus (EBV) nucleic acid 6.67 × 10^4^ copy/mL. Cytomegalovirus DNA were not detected. Bone marrow biopsy cytology, pathology and flow immunophenotyping suggested bone marrow metastasis from BL. Dexamethasone (10 mg qd), rituximab (500 mg, 375 mg/m^2^ body surface area), ATT, cardioprotection, hepatoprotection and alkalinization of urine were administered. Two days after the administration of rituximab infusion, the patient developed palpitations unbearable palpitations, followed by multi-organ damage and died 3 months later.

## 3. Discussion

Patients who receive long-term immunosuppressive medication have a much higher chance of developing opportunistic infections, among which TB is one of the most prevalent opportunistic pathogens.^[5]^ Immunosuppression causing low levels of T-lymphocyte responses results in a higher susceptibility to TB in transplant recipients. Low immune responses in KTRs often lead to atypical clinical symptoms of TB infection, low positive rate of tuberculin test, and multiple pathogenic infections, which poses a serious challenge for clinical diagnosis and treatment.

In clinical practice, KTRs who exhibit unexplained fever, lung CT findings of multiple nodular and patchy shadows and enlarged mediastinal lymph nodes, and suboptimal responses to traditional antibacterial and antiviral therapy should be warned that TB infection may be present. Further improvement of examination and definite diagnosis is crucial for disease regression. Prompt bronchoscopy alveolar lavage, brushing, biopsy, or thoracoscopic biopsy can assist doctors in determining a precise diagnosis. Besides, some studies demonstrated Interferon-γ release assay, Xpert Mycobacterium TB/rifampicin, and Metagenomic next-generation sequencing are new tests with high sensitivity and specificity, which are beneficial for early diagnosis of pulmonary TB.^[6,7]^

In this case, the patient clinical symptoms of TB were atypical (night-sweat only). The lung tissue and alveolar lavage fluid obtained were negative for antiacid staining, but the culture of TB in bronchoalveolar lavage fluid was positive 2 months later. Due to the delay of the culture results, we finally used the ATT after the consideration of a history of TB, TB genetic testing of lung tissue, lung histopathological and images results. Some researches had reported that previously treated (i.e., recurrent) TB cases account for approximately 7% to 8% of incident TB globally.^[8]^ Recurrence of TB disease after clinical cure/treatment completion of a previous episode may be due to endogenous reactivation of residual tuberculous bacilli from the original episode (referred to as relapse) or exogenous reinfection.^[8–10]^ Therefore, reinfection with TB is considered a high possibility. A potential risk factor of the patient may be the immunosuppressive therapy. Furthermore, the treatment of TB after renal transplantation requires comprehensive adjustment of immunosuppressive drugs and the standardized application of antitubercular agents, as well as close monitoring of liver and kidney functions and immunosuppressive concentrations.

After SOT or hematopoietic stem cell transplantation, PTLD, a heterogeneous proliferative disease of lymphoid tissue, can develop. Current studies suggest that the use of immunosuppressive drugs and EBV infection may be associated with the development of PTLD.^[11]^ Kidney transplantation is the most common type of transplantation, and when patients develop PTLD, it can significantly affect the prognosis. Our patient had many clinical features that put her at high risk of a poor outcome. First, she had late-onset of PTLD, 9 years after renal transplant. Relative to early-onset PTLD, late-onset PTLD is often associated with more monoclonal lesions and a worse prognosis.^[12,13]^ Second, she had BL, which is rare and more aggressive.

BL has 3 variants: endemic, sporadic and immunodeficiency-associated.^[14]^ Immunodeficiency-associated BL is commonly seen in patients with HIV infection, congenital immunodeficiency syndrome, or allogeneic transplants. A study investigated the incidence of BL in 203,557 solid organ recipients was 10.8 per 100,000 person-years.^[3]^ The risk of BL after SOT is 23 times higher than in the general population, peaking at 3 to 8 years after transplantation. Herein, BL-PTLD is probably a complication of a very prolonged immunosuppression. Transplantation at age < 18 years was reported to be an independent risk factor for BL-PTLD.^[3]^ And our patient had his first kidney transplant at the age of 13 years. Notably, BL was negatively associated with the treatment of azathioprine or corticosteroids, compared with other immunosuppressive drugs.^[3]^ Therefore, the use above drugs may reduce the possibility of BL after kidney transplantation.

Post-transplant BL usually presents with advanced clinical stage and systemic lymphadenopathy.^[15]^ One study has reported that 75% anatomic sites of BL were lymph nodes and 25% were extra nodal sites (16% abdominal, including the small or large intestine and liver, 4% in bone marrow, and 5% in miscellaneous sites).^[3]^ Clinical features of BL after renal transplantation depend on sites of invasion. When BL caused complete third nerve palsy with pupillary involvement, the clinic will be headache, left eye pain, diplopia, and complete ptosis.^[4]^ BL also can appear on the gingiva.^[16]^ In our case, the patient had BL detected in liver and bone marrow. BL evades the liver, and the patient presents with abdominal distension and decreased appetite. Pathologic findings of posttransplant BL were typical for Burkitt lymphoma. Genotypic and phenotypic aberrations were frequently seen in series of posttransplant BL.^[17]^ In posttransplant BL, c-MYC rearrangement is a consistent finding. In addition, other genetic changes, such as additional chromosomal abnormalities and p53 mutation, are frequently found,^[15]^ which is consistent with ours. Studies had demonstrated p53 was associated with poor prognosis in BL patients.

EBV was detected in 70% of patients with post-transplant BL.^[17]^ In addition, LDH and β2-MG are often present with varying degrees of elevation.^[18,19]^ Positive EBV nucleic acid and elevated indicators such as lactate dehydrogenase and β2-MG were seen during the course of the disease. A case report^[20]^ describes a 15-year-old boy, 3.5 years post-transplant with chronic EBV viremia, whose urinary CXCL10/Cr level increased acutely to 79.43 ng/mmol, 0.8 months prior to onset of BL. It means acute rise in urinary CXCL10/Cr was associated with onset of graft-associated BL. However, uncommon and potential sources of CXCL10 elevation needed to be highlighted in the future.

Currently, the common treatments of BL include the adjustment of immunosuppressive dosage, antiviral drugs, chemotherapy and targeted drug therapy.^[21]^ Targeted medications have gained popularity as first-line treatments in recent years due to their advantages of low side effects and excellent tolerance, which can also be used alone or in combination with cytotoxic drugs.^[11,22,23]^ In this case, the patient developed palpitations that were difficult to tolerate during the infusion of rituximab.

The patient’s condition had an insidious beginning and was progressing quickly, with multiple nodules spread throughout the body. The pathological examination results of endobronchial ultrasound-guided transbronchial needle biopsy of mediastinal lymph nodes suggested the possibility of TB infection. In addition, positive TB gene detection of lung specimens helped the diagnosis of pulmonary TB. At first, we considered that the systemic nodules were likely to be disseminated TB. After ATT, the patient’s clinical symptoms and signs improved, and the imaging showed that the pulmonary and hepatic nodules were reduced. During the process, the patient was maintained on low doses of hormones due to the need for anti-rejection in KTRs. Later, due to the appearance of symptoms of TB toxicity, the patient increased the dosage of hormones, which are important drugs for the treatment of lymphoma. So it was not possible to determine the effective proportion of both anti-TB and treatment of lymphoma in improving the patient’s condition. It took around 2 months from the time the patient’s many nodules were found to the time the diagnosis was confirmed. Therefore, in organ transplant patients, who present with multiple nodules and normal tumor markers, they should be alerted to the possibility of concurrent TB and PTLD, and perfect tests such as EBV, β-2-MG, LDH, γ-interferon release test and Xpert Mycobacterium TB/rifampicin test and perform early lesion site biopsy to clarify the diagnosis with a view to improving the prognosis.

## Author contributions

**Conceptualization:** Mu-Qing Yu, Shu-Yun Xu.

**Data curation:** Jian-Nan Hu, Chen Bao.

**Investigation:** Qian Liu, Chao Liu, Zi-Ling Li.

**Writing – original draft:** Jian-Nan Hu, Li-Juan Hua.

**Writing – review & editing:** Mu-Qing Yu, Xi Wang, Shuyun Xu.

## References

[1] Fang G Cheng NC Huang LL. The first report of co-existence of pulmonary tuberculosis and lung malignancy in a kidney transplant recipient: a case report and literature review. BMC Infect Dis. 2021;21:629. 34210287 10.1186/s12879-021-06350-x PMC8252204

[2] OʼRegan JA Prendeville S McCaughan JA. Posttransplant lymphoproliferative disorders in Irish renal transplant recipients: insights from a national observational study. Transplantation. 2017;101:657–63. 27214265 10.1097/TP.0000000000001201

[3] Mbulaiteye SM Clarke CA Morton LM. Burkitt lymphoma risk in U.S. solid organ transplant recipients. Am J Hematol. 2013;88:245–50. 23386365 10.1002/ajh.23385 PMC3608801

[4] Soyoral Y Dogan E Sayarlioglu H. Burkitt lymphoma in renal transplant recipient. Ren Fail. 2007;29:115–6. 17365922 10.1080/08860220601039544

[5] Cahuayme-Zuniga LJ Brust KB. Mycobacterial infections in patients with chronic kidney disease and kidney transplantation. Adv Chronic Kidney Dis. 2019;26:35–40. 30876615 10.1053/j.ackd.2018.09.004

[6] Meije Y Piersimoni C Torre-Cisneros J. Mycobacterial infections in solid organ transplant recipients. Clin Microbiol Infect. 2014;20(Suppl 7):89–101. 24707957 10.1111/1469-0691.12641

[7] Horne DJ Kohli M Zifodya JS. Xpert MTB/RIF and Xpert MTB/RIF Ultra for pulmonary tuberculosis and rifampicin resistance in adults. Cochrane Database Syst Rev. 2019;6:Cd009593. 31173647 10.1002/14651858.CD009593.pub4 PMC6555588

[8] Gan SH KhinMar KW Ang LW. Recurrent tuberculosis disease in Singapore. Open Forum Infect Dis. 2021;8:ofab340. 34307732 10.1093/ofid/ofab340 PMC8297698

[9] McIvor A Koornhof H Kana BD. Relapse, re-infection and mixed infections in tuberculosis disease. Pathog Dis. 2017;75. doi:10.1093/femspd/ftx020. 10.1093/femspd/ftx020 28334088

[10] Vega V Rodríguez S Van der Stuyft P. Recurrent TB: a systematic review and meta-analysis of the incidence rates and the proportions of relapses and reinfections. Thorax. 2021;76:494–502. 33547088 10.1136/thoraxjnl-2020-215449 PMC8225554

[11] Sprangers B Riella LV Dierickx D. Posttransplant lymphoproliferative disorder following kidney transplantation: a review. Am J Kidney Dis. 2021;78:272–81. 33774079 10.1053/j.ajkd.2021.01.015

[12] Quinlan SC Pfeiffer RM Morton LM. Risk factors for early-onset and late-onset post-transplant lymphoproliferative disorder in kidney recipients in the United States. Am J Hematol. 2011;86:206–9. 21264909 10.1002/ajh.21911 PMC3311225

[13] Luskin MR Heil DS Tan KS. The impact of EBV status on characteristics and outcomes of posttransplantation lymphoproliferative disorder. Am J Transplant. 2015;15:2665–73. 25988622 10.1111/ajt.13324 PMC5726526

[14] Crombie J LaCasce A. The treatment of Burkitt lymphoma in adults. Blood. 2021;137:743–50. 33171490 10.1182/blood.2019004099

[15] Gong JZ Stenzel TT Bennett ER. Burkitt lymphoma arising in organ transplant recipients: a clinicopathologic study of five cases. Am J Surg Pathol. 2003;27:818–27. 12766587 10.1097/00000478-200306000-00014

[16] Unholzer A Starz H Hirschsteiner O. [Gingival Burkitt lymphoma in a hepatitis C-positive renal transplant patient]. J Dtsch Dermatol Ges. 2005;3:46–51. 16353750

[17] Bobillo S Abrisqueta P Sánchez-González B.; Grupo Español de Linfomas/Trasplante Autólogo de Médula Ósea (GEL/TAMO cooperative group). Posttransplant monomorphic Burkitt’s lymphoma: clinical characteristics and outcome of a multicenter series. Ann Hematol. 2018;97:2417–24. 30116871 10.1007/s00277-018-3473-8

[18] Katz-Greenberg G Ghimire S Zhan T. Post-transplant lymphoproliferative disorders (PTLD)-from clinical to metabolic profiles – a single center experience and review of literature. Am J Cancer Res. 2021;11:4624–37. 34659910 PMC8493408

[19] Kim HD Cho H Kim S. Prognostic stratification of patients with Burkitt lymphoma using serum β2-microglobulin levels. Cancer Res Treat. 2021;53:847–56. 33332932 10.4143/crt.2020.1060 PMC8291169

[20] Suresh S Dix D Wang L. High urinary CXCL10/Cr with onset of Burkitt lymphoma in a pediatric kidney transplant recipient. Pediatr Transplant. 2022;26:e14354. 35869900 10.1111/petr.14354

[21] Al-Mansour Z Nelson BP Evens AM. Post-transplant lymphoproliferative disease (PTLD): risk factors, diagnosis, and current treatment strategies. Curr Hematol Malig Rep. 2013;8:173–83. 23737188 10.1007/s11899-013-0162-5 PMC4831913

[22] Deng W Wan Y Yu JQ. Pulmonary MALT Lymphoma has variable features on CT. Sci Rep. 2019;9:8657. 31209274 10.1038/s41598-019-45144-9 PMC6572828

[23] DiNardo CD Tsai DE. Treatment advances in posttransplant lymphoproliferative disease. Curr Opin Hematol. 2010;17:368–74. 20473161 10.1097/MOH.0b013e328339018c

